# Study of the Characteristics and Comprehensive Fuzzy Assessment of Indoor Air Chemical Contamination in Public Buildings

**DOI:** 10.3389/fpubh.2021.579299

**Published:** 2021-05-05

**Authors:** Xiguan Liang, Zhisheng Li, Huagang Zhang, Xinru Hong

**Affiliations:** School of Civil and Transportation Engineering, Guangdong University of Technology, Guangzhou, China

**Keywords:** fuzzy assessment, indoor pollutant concentration, air quality, correlation analysis, PM_2.5_

## Abstract

Quality-of-life is improving daily with continuous improvements in urban modernization, which necessitates more stringent requirements for indoor air quality. Fuzzy assessment enables us to obtain the grade of the evaluation object by compound calculation with the help of membership function and weight coefficient, overcoming the limitations of traditional methods applied to develop environmental quality indices. First, this study continuously measured SO_2_, O_3_, NO_2_, NO, CO, CO_2_, PM_10_, PM_2.5_, and other chemical pollutants during the daytime operating hours of a library and a canteen. We analyzed the concentration distributions of the particles in the air were discussed based on 31 different particle diameters. Finally, the experimental data in department store and waiting hall were analyzed by fuzzy evaluation, with the following results. (1) The library and canteen PM_10_ concentrations peaked at 07:45 in the morning and was elevated during the afternoon (48.9 and 59 μg/m^3^, respectively). (2) The Pearson correlation coefficient of the PM_10_ and PM_2.5_ concentrations in the library was 0.98. PM_10_ and SO_2_ in the canteen were negatively correlated, with a correlation coefficient of −0.65. PM_2.5_ and PM_1_ were always highly positively correlated. (3) The high concentration of particles in the library was associated with the small particle size range (0.25~0.45 μm). (4) By applying the fuzzy comprehensive evaluation method, the library grade evaluation was the highest level, and the waiting hall was the lowest. This study enhances our understanding of the indoor chemical contamination relationships for public buildings and highlights the urgent need for improving indoor air quality.

## Introduction

Indoor air pollution has become a hot topic because we realize that outdoor pollutants may infiltrate into indoor places and that these indoor areas may also have various sources of pollutants. An increasing number of people believe that indoor air pollution has an equal or even greater significant impact on human health compared with that of atmospheric environmental air quality (1). Depending on the geographic area, age, sex, work activities, and season, people spend ~87.5% of their day indoors (2). The number of German children spending a majority of their day indoors is ~75% (3), while the corresponding percentage of American adults is over 90% (4). Particulate matter concentrations and some indoor parameters may be only a fraction of those of outdoor pollutants, but this generally occurs only when there is no indoor source. When there is an indoor source, the indoor concentration of pollutants can exceed the outdoor concentration (5, 6). With the increasing frequency of hazy weather and the increasing degree of pollution, the issue of chemical pollutants has become increasingly prominent.

Differences in the airtightness of buildings may lead to significantly higher indoor concentrations of pollutants than outdoor concentrations, which may also be due to the full range of indoor emission sources (7). Indoor air quality presents one of the significant environmental risks to human health (8, 9). Studies have shown that exposure to indoor air pollutants can have a wide range of health effects. Ozone is an air pollutant with a multitude of potential adverse health effects. Hu et al. (10) found that indoor oxidizing gaseous pollutants, i.e., O_3_, had potential physical and chemical damage to objects indoors; moreover, ozone can also induce respiratory inflammation in healthy people and people with respiratory diseases such as asthma (11). Ozone is also associated with increased hospital admissions (12). Huang et al. suggested that indoor ozone <10 ppb was associated with cardiovascular responses in children (13). The impact of indoor exposure to particulate matter on health depends on the particle size, with particle sizes <1 μm having the greatest impact (14). PM_2.5_, PM_10_, and NO_2_ were found to increase wheezing and rhinitis symptoms among preschool children in China (15), and Asian schools presented higher levels of PM and PAHs than did European and American schools (16). Kidney damage induced by subchronic fine particulate matter exposure, especially exposure to PM_2.5_, may induce fibrosis and mesangial expansion (17). Children displayed significant associations between PM exposure and asthma, and in adults, PM_2.5_ was associated with asthma, bronchitis, and combined respiratory problems (18). In general, there is an association between children's health and exposure to ultrafine particles (UFPs), especially among children with respiratory diseases, who commonly experience alterations in inflammatory biomarkers and deterioration in lung function as a result of UFP exposure (19). Formaldehyde, which is a ubiquitous organic pollutant often found indoors, has recently been identified as a human carcinogen (20, 21). Long-term exposure to PM_2.5_ has been associated with an increased risk of new-onset depressive symptoms, while increased concentrations of nitric dioxide during summer are associated with the worsening of existing depressive conditions (22, 23). Exposure to low concentrations of NO_2_ increases the risk of premenopausal breast cancer (24–26), and we found evidence that long-term exposure to particulate matter and NO_2_ were linked with the development of heart failure (27). SO_2_ can change testicular structures and increase sperm malformation (28, 29). Volatile Organic Compounds (VOCs) are widely reported in public buildings. The World Health Organization, the Committee on Indoor Air Pollution Japan, the US Environmental Protection Agency and Public Health England have assessed the evidence and listed the potential health effects of VOCs, including irritation of the eyes and respiratory tract, allergies and asthma, central nervous system symptoms, liver and kidney damage, as well as cancer risks (30, 31). Therefore, a variety of pollutants, not only those causing discomfort, should be considered when assessing indoor air quality.

However, there is currently a lack of comprehensive indicators for use in indoor air quality assessment. We aim to apply an indoor air quality assessment program based on fuzzy analysis to improve on previous methods of indoor air quality assessment. Many scholars have studied indoor air quality assessment methods, and their mathematical models were formed using a variety of methods, such as objective and subjective evaluations, as well as integrated and unified assessments. Some scholars have studied the decipol evaluation method and air quality index method (32, 33), but the methods could not quantitatively express the perceived air quality or dynamically track pollutants, respectively. The indoor air pollutant gray clustering model is proposed to simplify the evaluation of indoor air quality, but it is impossible to adjust the critical evaluation criteria sequence (34). Office buildings in Brisbane, the capital city of Queensland, Australia, have no obvious pollution indoors, yet there is a strong correlation between particle concentrations of indoor and outdoor pollutants there (35). Formaldehyde and toluene are major indoor pollutants of concern in newly constructed apartment buildings (36). However, research on these pollutants does not consider the chemical pollutant effect on the relationship between indoor and outdoor concentrations of PM_2.5_. In addition, these studies lack high-time resolution monitoring data, so there is still room for improvement.

This article is based on a study of a library and canteen in two public buildings in Guangzhou, China, where the indoor chemical pollutant concentrations were measured with the windows closed and with the windows open for ventilation in the spring. We analyzed the air quality according to the indoor and outdoor PM_2.5_ and PM_10_ concentrations and the changing trend of each pollutant and calculated the correlation between the chemical pollutants, as well as the particle size distribution of the indoor particulate matter. Statistics are presented for the concentrations corresponding to three main occupational health concerns from indoor pollutants, including Inhalable, Thoracic, and Alveoli categories. Additionally, the experimental data in department store and waiting hall were analyzed by fuzzy evaluation. These studies on the effects of indoor and outdoor PM_2.5_ levels provide a basis and method for reference, as well as a reference for long-term health assessments for practitioners in the area.

## Materials and Methods

### Sampling Sites and Time

We selected a library (113.4019°E, 23.0439°N), a canteen (113.3904°E, 23.0485°N), a waiting hall (113.3502°E, 23.1761°N), and a department store (113.3983°E, 23.0688°N) in China as the test objects. The library, the canteen, and the department store are located on the campus of a university in Panyu district of Guangzhou. The waiting hall, located in Tianhe District, is intended as a terminal station for a number of bus passengers. The field experiment was conducted for a total of 4 days, on April 15, 16, 24, and 25, 2019. In the first-two-day experiment, the test duration for each kind of ventilation was ~8 h in the daytime after opening. The specific timetable of the field experiment is shown in Table 1. It is worth mentioning that in the last two measurements of April 24 and 25, to verify the fuzzy assessment, we focused on the peak hours of public buildings. Therefore, the concentration it measured will only be used to carry out feasibility analysis of the fuzzy comprehensive evaluation model.

**Table 1:** The timetable of the field experiment.

**Date**	**Site**	**Time**	**Ventilation**	**Area**
2019.04.15	Library	08:00~16:30	Mechanical	112.60 m^2^
2019.04.16	Canteen	07:00~16:00		101.25 m^2^
2019.04.24	Waiting hall	11:30~12:30		150.00 m^2^
2019.04.25	Department store	11:30~12:30		162.50 m^2^

The indoor and outdoor PM_2.5_ and other pollutants were measured in the centralized central air conditioning. During the test period, there were no distinct outdoor PM_2.5_ or other pollution sources, such as construction projects, rain or hazy weather. The information about sampling sites is listed in Table 1. The internal and external windows of the test area were closed throughout the whole process, and the indoor area was equipped with a VRF system. Data from each site was dynamically recorded by testers. All indoor rooms were non-smoking rooms.

### Sampling Method

**Figure 1:**
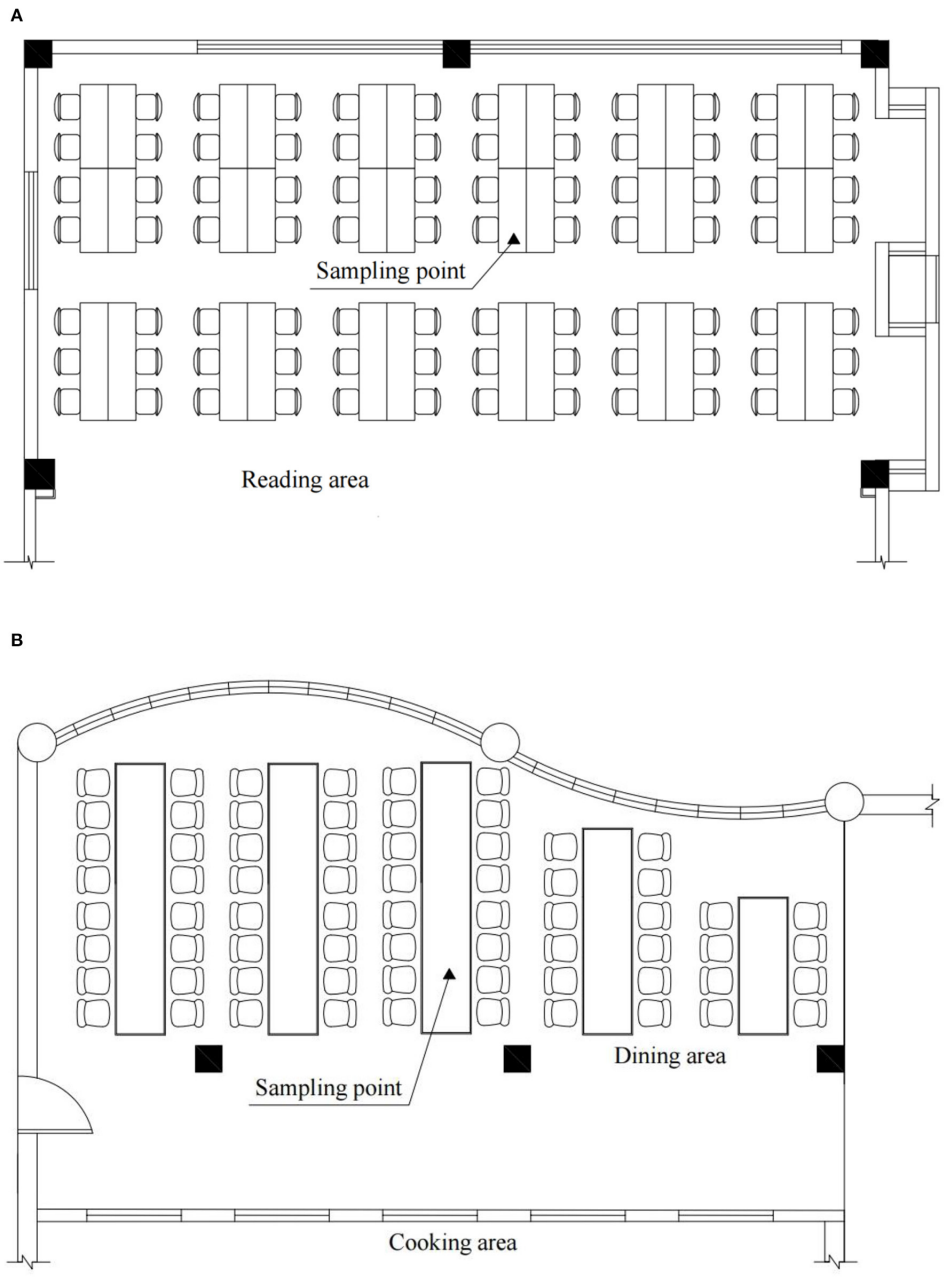
Indoor measuring points in the library **(A)** and canteen **(B)**.

**Figure 2:**
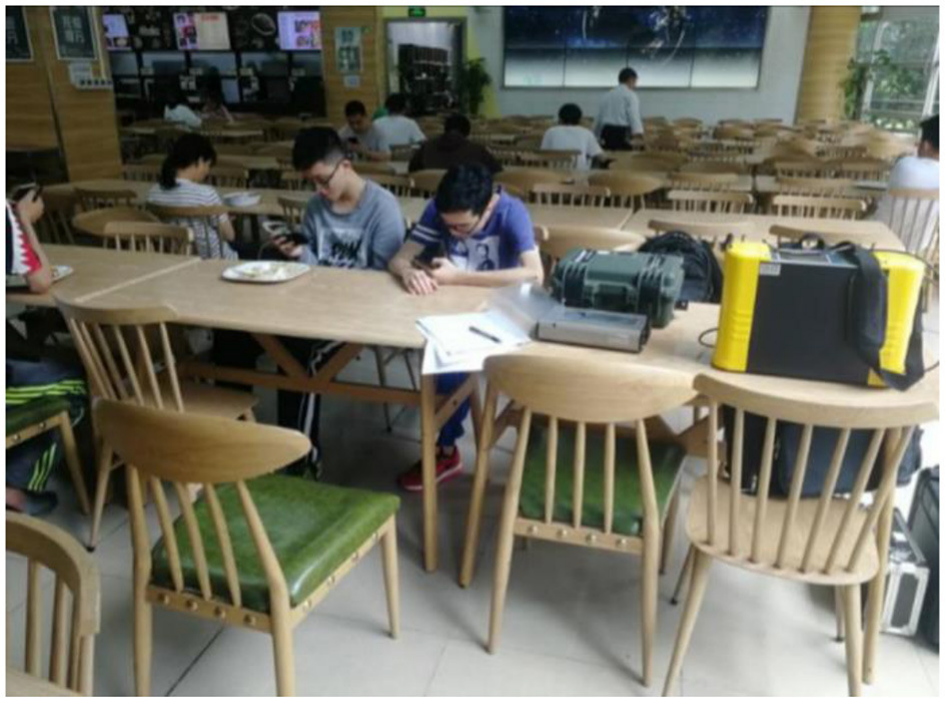
Field layout of the indoor measuring instruments. Data from both sites were dynamically recorded by testers.

According to the relevant provisions of the indoor air quality standard (UB/T 18883-2002), the sampling points for monitoring the concentrations of chemical pollutants in the library were arranged on a desk table on the 4th floor (8 floors in total), 4.3 m away from the exterior window of the building and 1.0 m high. The sampling points for measurements of the canteen were arranged in the customer dining area on the first floor and close to the outer pane of the canteen. The measuring point was 1.0 m from the ground and 3.2 m from the external window. The specific layout is shown in Figures 1, 2. We also chose the central location of the other two buildings as the measuring point.

### Sampling Instrument

The mass concentrations of indoor particles were recorded by a portable laser aerosol diameter spectrometer (11-R, GRIMM Aerosol Technique Airing Inc., Germany) at an interval of 1 min in real time. After the test, the measured data were uploaded to the connected workstation computer via a serial port to a USB data line. SO_2_, O_3_, NO_2_, NO, and CO were recorded by the air quality monitoring system (HIM-6000, Haz-Scanner Inc., U.S.A) at an interval of 5 min. A portable infrared component gas analyzer (Gasboard-3100P, Cubic-Ruiyi Inc., China) assessed CO_2_ and CH_4_ at an interval range of 5 min in real time. The hourly outdoor pollutant data were monitored in real time by the nearest Chisha monitoring station. The data were obtained from the real-time air quality release system issued by the Guangzhou Environmental Monitoring Center station, and we obtained the changes in pollutant concentrations at each monitoring point through a summary of statistics. The measurement ranges and accuracies are listed in Table 2.

**Table 2:** The measurement ranges and accuracies.

**Contamination**	**Instrument model**	**Range**	**Accuracy**	**Resolution**	**Manufacturer**	**Producing area**
SO_2_	HIM-6000	0~5000 ppb	±5 ppb	5 min	Haz-Scanner	U.S.A.
NO_2_	HIM-6000	0~5000 ppb	±5 ppb	5 min	Haz-Scanner	U.S.A.
NO	HIM-6000	0~25 ppm	±10 ppb	5 min	Haz-Scanner	U.S.A.
CO	HIM-6000	0~100 ppm	±0.01 ppm	5 min	Haz-Scanner	U.S.A.
CO_2_	Gasboard-3100P	0~40%	±1%FP	5 min	Cubic-Ruiyi	China
CH_4_	Gasboard-3100P	0~75%	±1%FP	5 min	Cubic-Ruiyi	China
O_3_	HIM-6000	0~150 ppb	±0.1 ppm	5 min	Haz-Scanner	U.S.A.
PM_2.5_	11-R	0.1 μg/m^3^ ~ 100 mg/m^3^	±3%	1 min	GRIMM	Germany
PM_10_	11-R	0.1 μg/m^3^ ~ 100 mg/m^3^	±3%	1 min	GRIMM	Germany
PM_1_	11-R	0.1 μg/m^3^ ~ 100 mg/m^3^	±3%	1 min	GRIMM	Germany

### Instrument Calibration

Compared with standard equipment, the measurement data of the portable sensor often has some linear error. Therefore, the portable sensor needs to be measured in the same place for a long time with standard equipment. Finally, the measurement results of the portable device are compared with the measurement results of standard equipment. In relation to data quality control, all the portable monitors were first calibrated before leaving the factory, and further estimations were made using standard methods prior to this study. Data calibration was first conducted at the Guangdong University of Technology (GDUT) on April 9, 2019. Calibration checks since then have been routinely performed before and after each run.

### Processing Measurement Data

#### Rejection of Outliers

Some sudden human activities (such as vehicles operating or suddenly starting near the testing area) often lead to abnormally high values of pollutant samples in dining areas or reading rooms (1, 37, 38), which may distort real pollution trends or aggravate typical-source pollution levels. The performance of the running coefficient of variation method was more convincing than those of other methods, making it an excellent choice for outlier sample testing and for elimination in air quality detection (39, 40). With this study's primary focus on indoor air pollution trend analysis to characterize general air quality trends. Theoretically, isolation of indoor air pollution trends may be simplified by site selection in an outdoor environment where roadways surrounding have no external human interference. However, such ideal conditions are rare and often studies need to consider these random events. Therefore, the pollutant indicators closely related to temporary anthropogenic emissions and the more sensitive pollutants needed to be determined. PM_2.5_ was selected as the reference in this paper. Next, based on the particle sample sequence of 1-min resolution, the 5-min slip variation coefficient of single-sample data was calculated. The calculation formula is as follows:

(1) $$\begin{array}{l} RCOV_{\text{s}}=\frac{\sqrt{\frac{1}{5}\sum_{i=s\text{-}2}^{i=s+2}{\left(a_{\text{i}}-\bar{a}\right)}^{2}}}{{\bar{a}}_{all}} \end{array}$$

where *RCOV*_s_ is the 5 min running variation coefficient of the *s*^th^ measured PM_2.5_ data; *a*_i_ is the *i*^th^ measured PM_2.5_ data; $\bar{a}$ is the mean value of the *s*^th^ measured PM_2.5_ data and the two adjacent samples collected before and after; and ${\bar{a}}_{\text{all}}$ is the mean value of all measured PM_2.5_ data in a one-day experiment. The 99^th^ quantile of the 5-min sliding coefficient of variation of all PM_2.5_ data was taken as the threshold, and measured PM_2.5_ data larger than this threshold were regarded as data of abnormally high values and were eliminated along with the two adjacent PM_2.5_ data collected before and after.

Taking PM_2.5_ as an example, the library involved a continuous daytime measurement lasting ~8 hours (the total number of samples with 1-min resolution was 526). In each experiment, samples with abnormally high values identified by the *RCOV* (the 99^th^ quantile was ~0.1269) method accounted for 0.56% of the total samples at 08:14~08:16. By combining these data with the outdoor data (Figure A1), we observed that the particle mass concentration at the monitoring site of Chisha also fluctuated slightly at this time. The test day was a working day, and the east wind prevailed during the season. The library was located on the southwestern side of a specific intersection, which experienced frequent working conditions of motor vehicles during the morning rush hour when teachers, students and staff of the school commuted to work. Such temporary and complex peripheral environmental factors were often accompanied by abnormally high-value PM_2.5_ samples during the measurement process.

#### Dynamic Correction

In general, dynamic testing experiments with specific functions or polynomial fitting may be used to process data if certain and uncertain factors need to be distinguished. However, sometimes, it is difficult to know the functional form of measured and complex test data, or it is difficult to know which polynomial to choose. Occasionally, we need to eliminate the random fluctuation in dynamic test data. It is not necessary to express its change rule in the form of some function or as the sum of some features, but it is necessary to reveal its definite change rule according to the value of the point function (41–44). It is also tricky to directly compare and process data from instruments with different time resolutions, so dynamic correction is required.

Based on previous studies, a relatively ideal correction method based on a time series, a 5-point equal-weight smooth-thin plate spline regression method (5S-TPS), was proposed, and the measured data from two the locations were used in the data processing (39, 42). This method was divided into three steps. (1) Along the full length of *N* data, the original pollution samples with the resolution of 1 min were processed with a 5-min moving average. Thus, five adjacent data points were continuously measured one by one to determine the arithmetic average. (2) The moving average results were divided into equal parts according to the specified time window (such as 5 min), and the positions of the specific time nodes of pollutant concentrations were identified in each corresponding window to eliminate random errors and synthesize time resolutions matching those of the other instruments. (3) Thin-plate spline regression was used to conduct smoothing fitting for the sample of the sliding mean value of the pollutant concentration at the specified time node obtained in the previous step.

#### Correlation Analysis

After the dynamic correction described above, the data with a time resolution of 1 min were compared with the data with a resolution of 5 min at the same time-scale interval. Here, the Pearson correlation coefficient *R*_p_ was used to judge the correlation between the pairwise measured parameters of each chemical pollutant. The calculation formula is as follows:

(2) $$\begin{array}{l} R_{\text{p}}\;=\;\frac{\overset{n}{\underset{i=1}{\sum }}\left({\text{x}}_{\text{i}}-\bar{x}\right)\left(y_{\text{i}}-ȳ\right)}{\sqrt{\overset{n}{\underset{i=1}{\sum }}{\left(x_{\text{i}}-\bar{x}\right)}^{2}}\cdot \sqrt{\overset{n}{\underset{i=1}{\sum }}{\left(y_{\text{i}}-ȳ\right)}^{2}}} \end{array}$$

where *x*_i_ and *y*_i_ (*i* = 1, 2, 3…) are the sample values and $\bar{x}$ and $\bar{y}$ are the sample mean values. The value range of the correlation coefficient is | *R*_p_ | ≤ 1, and the closer the absolute value is to 1, the greater is the correlation between variables *x* and *y*. Generally, | *R*_p_ |>0.8 means highly correlated, and | *R*_p_ |<0.3 means weakly correlated. This portion is implemented using the R programming language operation platform RStudio.

### Description of Fuzzy Evaluation

Fuzzy Comprehensive Evaluation is a famous method and has extensively been applied in many fields including wind farm investments, masonry structure safety, teaching performance, and landslide susceptibility (45–48). It is able to help the researchers to find out the most important factors that should be prioritized by using mathematics. It has been proved that the combined application of fuzzy mathematical method on the air assessment is not only objective but also quantitative (49). A fuzzy inference system is quite appropriate for subjective issues like environmental conditions, because it can appropriately classify them (especially in the case of boundary levels that are failed to be sensitively classified by conventional methods), help a balance between different and sometimes contradictory observations be achieved, and finally help subjective and non-quantitative issues like air quality assessment be well-classified and quantified (50).

The discontinuous distribution of the air pollution grading index concentration was inconsistent with the continuous distribution of the indoor air quality index. Given the inherent complexity, risks and uncertainties of AQI and the diversity of sites, this paper aims to introduce a novel assessment framework for contamination using fuzzy assessment as a recent approach dealing with uncertainties in in the field of indoor air quality evaluation. Introducing the fuzzy mathematics principle to this study of the public space involved using monitoring data to create the corresponding fuzzy mathematical model. The model was applied to six evaluation objects for the indoor environmental air quality. The fuzzy comprehensive evaluation was based on monitoring of the indoor air pollution, and personnel from these public workplace buildings were provided with the health assessment to provide a scientific basis for breathing. The prevalence of sick building syndrome has caused people to focus increasing attention on the problem of indoor air quality. Therefore, among the many methods of indoor air quality evaluation, the fuzzy mathematics theory is a highly recognized method (51–53).

**Figure 3:**
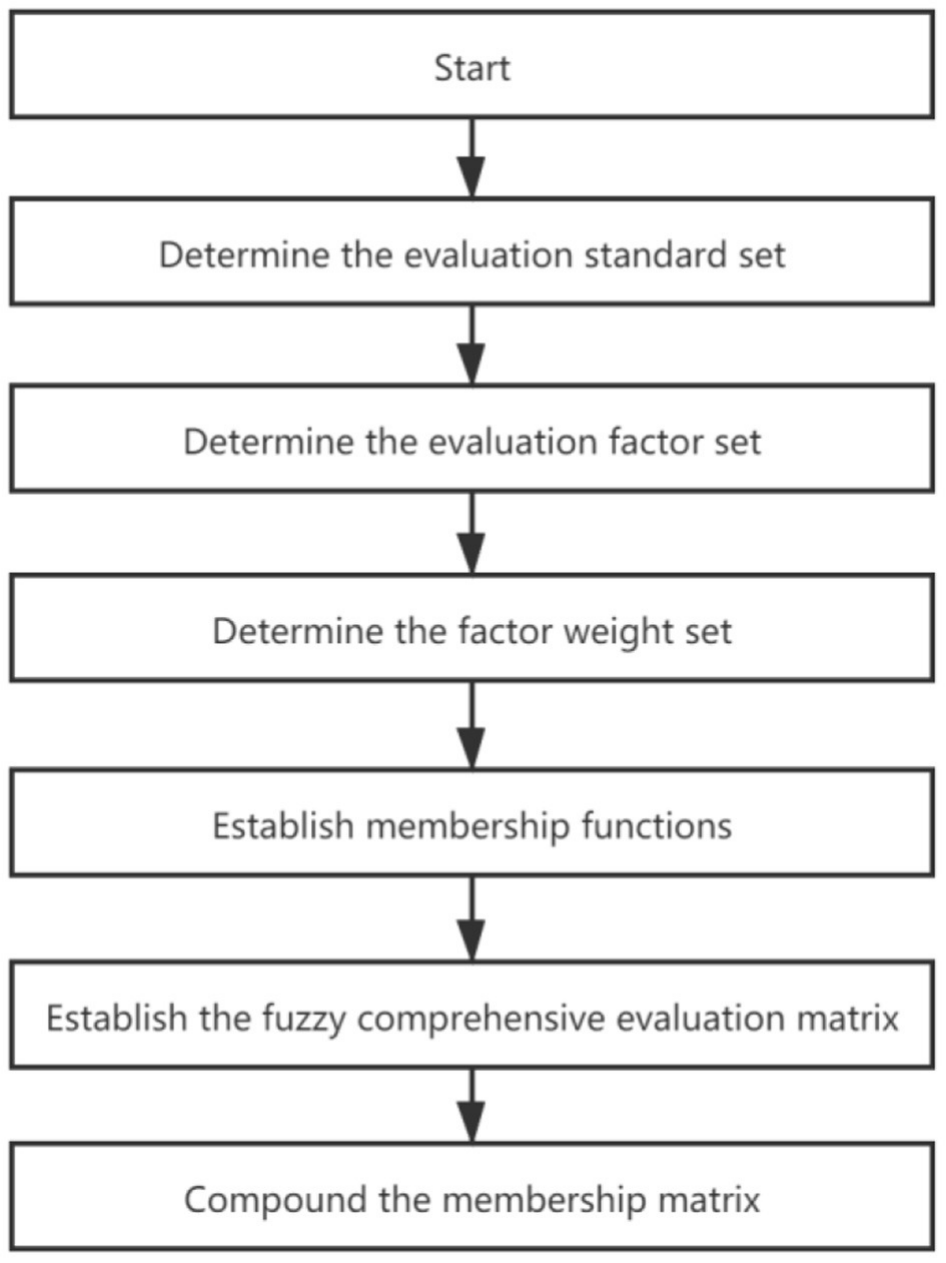
Schematic of fuzzy comprehensive evaluation for indoor air quality.

The specific evaluation steps (Figure 3) were as follows:

1) Determine the evaluation standard set of the research object *V*={*V*_1_, *V*_2_…, *V*_m_} according to the improved indoor evaluation index (54), and combine the set with the actual situation of the indoor air pollution, with each degree factor of environmental air pollution divided into five levels, namely, I (clean), II (light pollution), III (moderate pollution), IV (heavier pollution), and V (polluted).

2) Determine the evaluation factor set of the research object *U*={*U*_1_, *U*_2_…, *U*_n_}. According to a comprehensive analysis of various indicators affecting the environmental air quality (52, 55), primary pollutants in the air include the following six items: PM_2.5_, PM_10_, CO, O_3_, SO_2_, and NO_2_. Therefore, the established evaluation factor set is *U*={PM_2.5_, PM_10_, CO, O_3_, SO_2_, NO_2_}.

3) Determine the factor weight set. We studied the medical literature and rearranged the weight coefficients of pollution parameters affecting the indoor air quality according to the degree of influence of various air chemical pollutants on human health (49). They were PM_2.5_(0.25), PM_10_(0.2), CO(0.2), O_3_(0.15), SO_2_(0.1), and NO_2_(0.1); thus, the factor weight set is *A*=[*a*_1_, *a*_2_, …, *a*_5_]=[0.25, 0.2, 0.2, 0.15, 0.1, 0.1].

4) Establish membership functions. A membership function is a curve with different possible shapes (for example, trapezoidal, triangular, or gaussian) that shows how each point in the input space is related to membership value between 0 and 1 (50). According to the above evaluation factor set and evaluation standard set, combined with the actual situation of the research problem, this paper established the membership function *r*_ij_ of each pollution factor for each level standard in the form of a reduced half trapezoid function, and the specific calculation formula is as follows:

For level I, i.e., *j* = 1, the membership function expression is:

(3) $$\begin{array}{l} r_{1i}=\{\begin{array}{l} 1,u_{i}⩽s_{1}\\ \frac{s_{2}-u_{i}}{s_{2}-s_{1}},s_{1}<u_{i}<s_{2}\\ 0,u_{i}⩾s_{2} \end{array} \end{array}$$

For level II - IV, i.e., *j* =2, …, *m*−1, the membership function expression is:

(4) $$\begin{array}{l} r_{ji}\text{=}\{\begin{array}{l} 0,u_{i}⩽s_{j-1}\\ \frac{u_{i}-s_{j-1}}{s_{j}-s_{j-1}},s_{j-1}<u_{i}<s_{j}\\ \frac{s_{j+1}-u_{i}}{s_{j+1}-s_{j}},s_{j}<u_{i}<s_{j+1}\\ 0,u_{i}⩾s_{j+1} \end{array}(j=2,\ldots ,m-1) \end{array}$$

For level IV, i.e., *j* = *m* = 5, the membership function expression is:

(5) $$\begin{array}{l} r_{jm}=\{\begin{array}{l} 0,u_{i}⩽s_{m-1}\\ \frac{u_{i}-s_{m-1}}{s_{m}-s_{m-1}},s_{m-1}<u_{i}<s_{m}\\ 1,u_{i}⩾s_{m} \end{array}\;,m=5 \end{array}$$

In the above piecewise expression of the function, *i* represents an evaluation factor in the evaluation factor set *U*; *j* represents an evaluation standard in the evaluation standard set *V*; *u*_i_ represents the measured pollutant concentration value at a particular place of the *i*^th^ evaluation factor; and *s*_j_ represents the concentration limit of the *j*^th^ grade of the *i*^th^ evaluation factor.

5) Establish the fuzzy comprehensive evaluation matrix. The monitoring values of the pollutants were substituted into the corresponding membership function expression, and the membership degree of each evaluation factor to each grade was calculated, forming the membership degree set of each pollution factor. Namely, the single-factor evaluation of the *V*i factor *R*_i_= (*r*_i1_, *r*_i2_…, *r*_in_)^T^ was a fuzzy subset, in which *r*_in_ represents the membership degree of the *i*^th^ factor for the *j* grade, and the evaluation matrix of all factors is *R* = (*R*_1_ *R*_2_… *R*_n_).

6) Compound the membership matrix *R*, which is obtained above, and the weight matrix *A* to obtain the comprehensive evaluation vector *B*, that is, *B* = *A*·*R* = (*b*_1_, …, *b*_m_). In traditional fuzzy mathematical theory, *b*_k_ is assumed to be *b*_j_ (*j* = 1) according to the maximum membership principle, and it can be concluded that the indoor air quality belongs to grade *k*.

(6) $$\begin{array}{l} B=\left[a_{1}\;a_{2}\ldots a_{\text{m}}\right]\;\left[\begin{array}{cccc} r_{11} & r_{12} & \ldots & r_{\text{ln}}\\ r_{21} & r_{22} & \ldots & r_{2\text{n}}\\ \ldots & \ldots & \ldots & \ldots \\ r_{\text{m1}} & r_{\text{m}2} & \ldots & r_{\text{mn}} \end{array}\right]=\left(b_{1}\;b_{2}\ldots b_{\text{n}}\right) \end{array}$$

## Results and Discussion

### Overall Characteristics of Each Pollutant During the Measured Period

**Figure 4:**
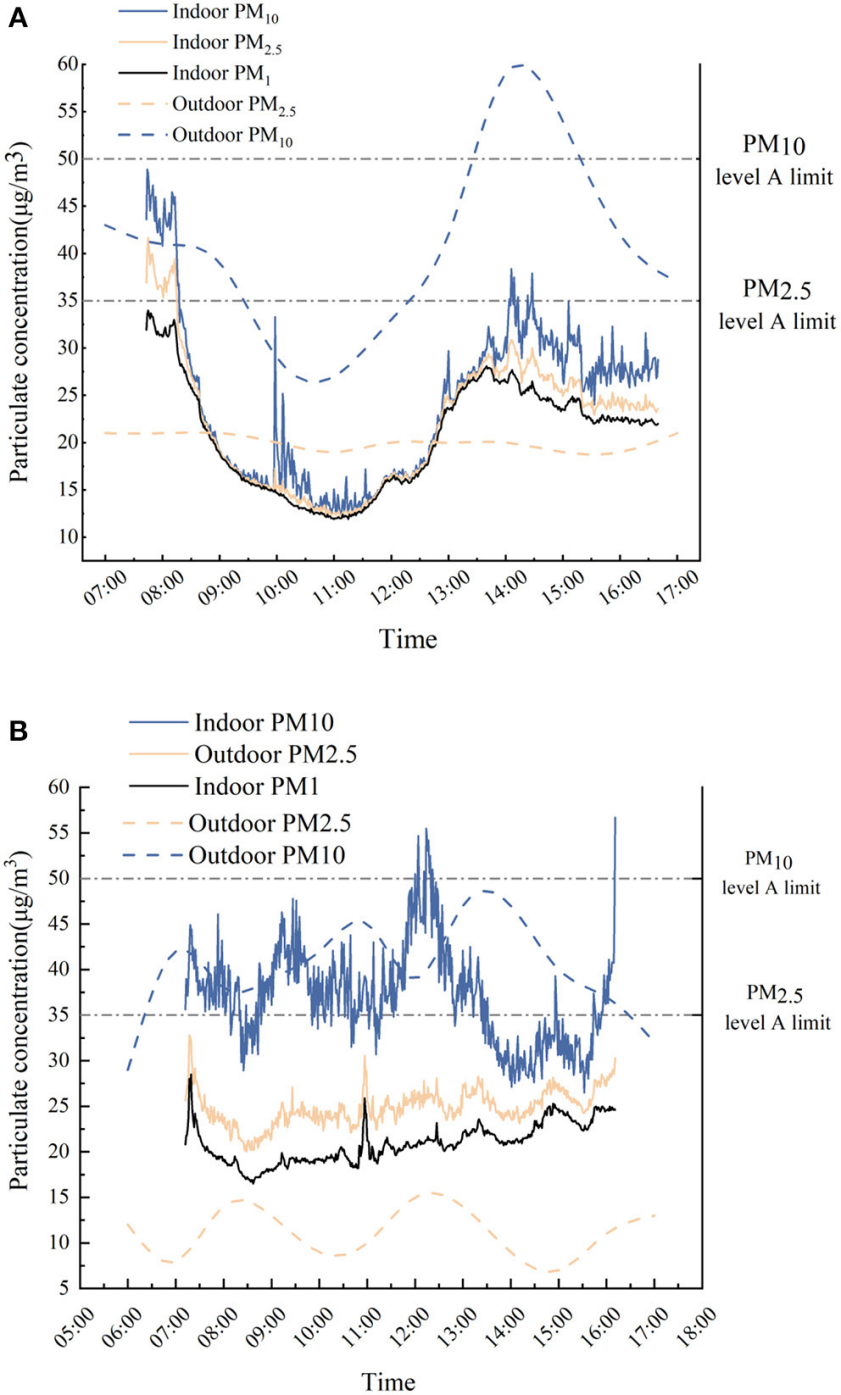
Hourly variation of PM_1_-PM_2.5_-PM_10_ inside and outside of the library **(A)** and canteen **(B)** based on the 5S-TPS method.

Guangzhou is located in the region with a hot summer and warm winter, one of the five climate zones of China. During the spring measurements, the weather was clear, and there was no cloudy rain or haze. According to the ambient air quality standard (GB 3095-2012), during the test, PM_2.5_ concentrations of 35 μg/m^3^ and PM_10_ concentrations of 50 μg/m^3^ could be used as the particulate level A judgment standard. For the library, the indoor air concentrations of PM_2.5_ and PM_10_ could achieve level A standards. Most of the time, before 08:15, the distribution of PM_2.5_ concentrations was at level B, and during the afternoon session (14:00 to 15:00), the indoor PM_10_ concentrations were at level B, while the outdoor PM_10_ concentrations were at level A. It can be seen from Figure 4 that there was an apparent synchronism between the indoor particulate matter and outdoor particulate matter in the library. PM_2.5_ and PM_10_ both showed a trend of first decreasing, then increasing and finally decreasing during the test. Surprisingly, the timing of the peak of the particulate matter was inconsistent. Taking PM_10_ as an example, the peak value in the library was at ~07:45 in the morning (48.9 μg/m^3^), while the peak value in the library was at ~14:00 (59 μg/m^3^) in the afternoon. When the public building was open, positive pressure formed in the room. Furthermore, the air conditioner was turned on. Thus, the permeability of particles leads to a difference in concentration between the inside and outside areas. The overall outdoor PM_2.5_ level did not change much, but the indoor PM_2.5_ concentration was higher than the outdoor concentration during the period from 9:00 to 13:00.

Most of the time, the concentrations of PM_2.5_ and PM_10_ in the air of the canteen could reach standard level A, with PM_2.5_ concentrations at level A in the daytime, while at noon (12:00–12:30), the indoor PM_10_ concentration could reach level B (Figure 4). The pollution from cooking combustion sources had certain relevance to these data. Unlike the library, the canteen showed inconsistent indoor and outdoor particulates due to the existence of internal pollution sources. The changing trends of indoor PM_2.5_ and PM_1_ were the same, but the concentration of PM_10_ changed sharply and was higher than the outdoor value at many times. PM_1_ concentrations peaked at ~07:19 am (28.5 μg/m^3^) in the canteen, PM_2.5_ concentrations peaked at ~07:17 am (32.8 μg/m^3^), and PM_10_ concentrations peaked at ~16:11 P.M. (56.7 μg/m^3^). The PM_2.5_ concentration in the canteen reached extreme values during the cooking periods before breakfast, lunch and dinner, with concentrations of 32.5, 30.6, and 30.3 μg/m^3^, respectively. Cooking oil fumes contain massive particulate matter according to Du et al. (56), which can escalate the concentrations at meal times.

The primary indoor and outdoor chemical pollutants were inconsistent. The main indoor pollutant was particulate matter, while the primary outdoor pollutant was CO. NO in the library fluctuated sharply within the range of 0~10 ppb, while SO_2_, O_3_ and NO_2_ were below 14 ppb. Taking 09:00 am as an example, the I/O ratio of CO was 1.25, the I/O ratio of NO_2_ was 0.11, the I/O ratio of O_3_ was 1.43, and the I/O ratio of SO_2_ was 1.07 (Figure A2). These amounts were still higher than the outdoor concentrations, although the indoor chemical pollutants did not exceed the standard limits.

Figure A3 and Figure A4 in appendix show the hourly variations in the percentages of 6 gases in the two locations. The variation trend of CO produced values that remained almost the same, but the concentration of CO_2_ gradually accumulated in the library, which may have been related to the building tightness and the increase in personnel. CO_2_ in canteen reached two peaks during the morning. At noon, the bulk concentration exceeded the daily value of 0.03% at most moments, and the maximum value reached 0.19% at 13:38 at noon. The volume concentration of CH_4_ was always below 0.3%, the volume concentration of C_n_H_m_ was almost 0%, and the difference between H_2_ and O_2_ was not significant.

### Effects of Indoor Air Chemical Pollutants

**Figure 5:**
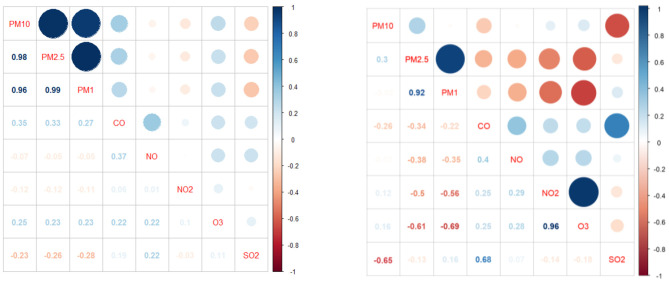
Distribution of the correlation coefficients of 8 contaminations in the library (**Left**) and canteen (**Right**).

According to the above analysis, the trends of different pollutants in different indoor environments were different. To further explore the relationships between the pollutants, the Pearson correlation coefficient *R*_p_ was used to judge the correlation between the pairings of the measured parameters. The results are shown in Figure 5. The main diagonal shows the categories of pollutants listed, and the size of the circle in the upper right indicates the correlation between the two pollutants. The larger is the graph, the stronger is the relationship; the darker is the blue color, the stronger is the positive correlation; and the darker is the red color, the stronger is the negative correlation. The value in the lower-left region is the Pearson correlation coefficient between the pairings, and the color depth also corresponds to the graphic meaning. In the library, PM_10_, PM_2.5_, and PM_1_ had a strong positive correlation (the correlation between PM_10_ and PM_2.5_ reached 0.98; 0.99 was for PM_2.5_ and PM_1_; and 0.96 was for PM_10_ and PM_1_). SO_2_ showed a weak negative correlation with particulate matter, with correlation coefficients of −0.23(PM_10_), −0.26(PM_2.5_), and −0.28(PM_1_). In the canteen, O_3_ and NO_2_ had the strongest positive correlation, with a coefficient of 0.96. The correlation coefficient between PM_2.5_ and PM_1_ was 0.92. Due to the existence of internal pollution sources, the concentration of PM_10_ was relatively high and not synchronized with those of other particles, and its correlation coefficient with PM_1_ was only 0.02. At this time, PM_10_ and SO_2_ were negatively correlated with each other, and the correlation coefficient was ~−0.65. PM_1_ and O_3_ also had a highly negative correlation, with a value of −0.69. Although the Pearson correlation coefficients of the same chemical pollutants in different public buildings varied greatly, PM_2.5_ and PM_1_ were always highly positively correlated, O_3_ in the library was weakly positively associated with other pollutants, and PM_2.5_ in the canteen was almost negatively correlated with other contaminants, and PM_1_ has the same characteristics.

### Distribution of Indoor Air Particles

**Figure 6:**
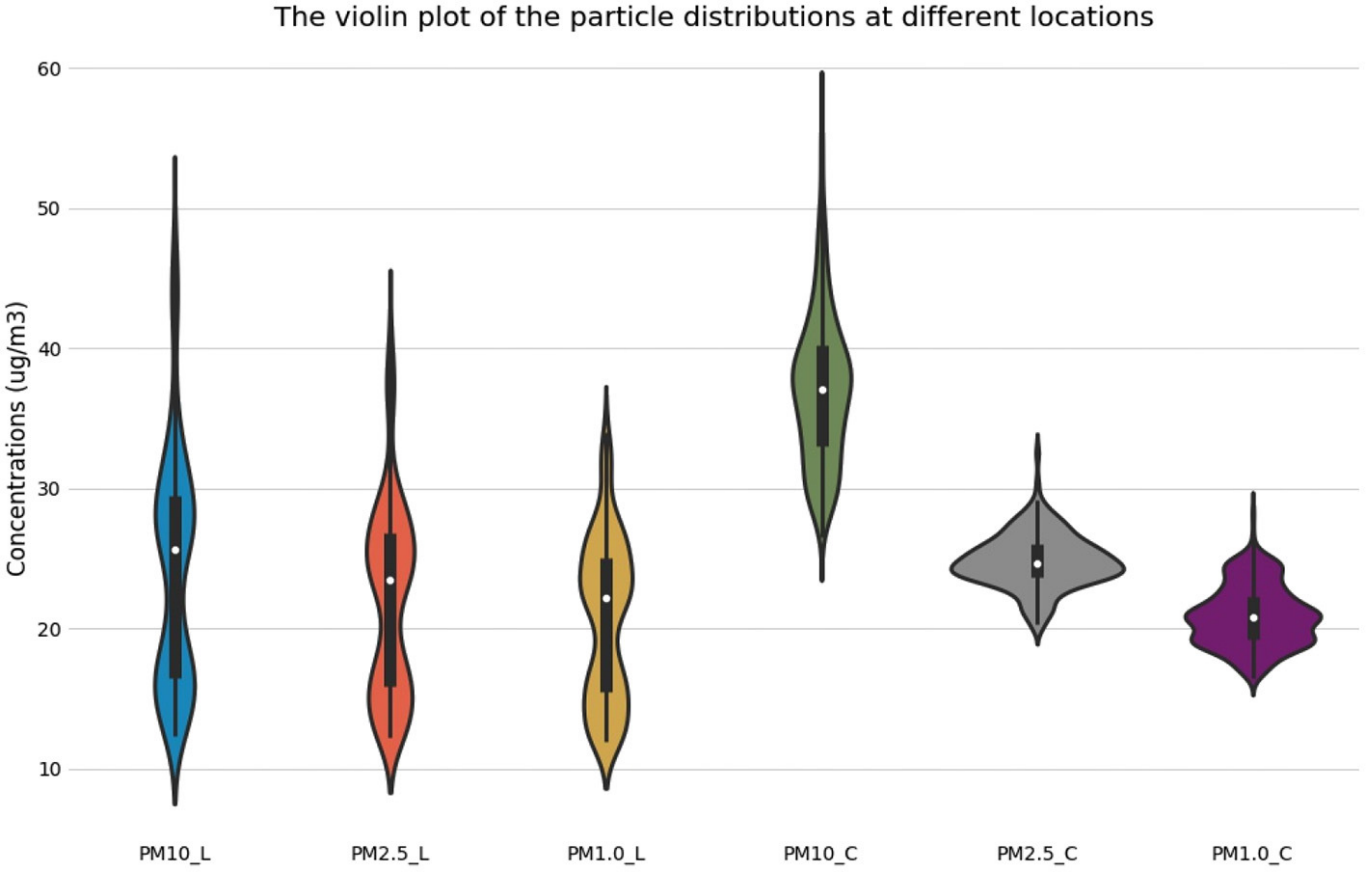
The violin plot of the particle distributions at different locations.

For the two locations, statistical tests were performed for the three kinds of particle size distributions in the mass concentrations of the particulate matter (Figure 6). Different concentrations of the object operation platform were tested, and using the R programming language RStudio (Version 1.2.1335) and an integrated development environment, a summary was plotted as a violin plot. The violin plot combined the boxplot and density figure characteristics to avoid hiding important details about the data distribution and could reflect the dispersion density and distribution of each interval period. The outer shape of the filled color block in the figure is the kernel density estimation, which was used to estimate the unknown density function in the probability theory. In one set of tests, the more times the concentration of a certain x-coordinate appeared, the larger was the y-coordinate value of the reaction on the graph, and the more concentrated was the whole graph. The data of the 95% sample values could be statistically analyzed in the violin plot using the R programming language. The results showed that the plot of the concentrations of particulate matter in the library was gourd-shaped, and the data in the middle and bottom areas were relatively concentrated. Among them, the highest concentrations of PM_10_ were 26.5 μg/m^3^ and 16.6 μg/m^3^, with each accounting for 1.68% of the total data. The mass concentrations of PM_2.5_ were 24.0 μg/m^3^, accounting for 2.34%, and 16.2 μg/m^3^, accounting for 1.49%. PM_1_ accounted for 2.98% and was present at 22.3 μg/m^3^. The distributions of the three particles were similar. The mass concentrations of particulate matter in the canteen were distributed in a spindle shape. The mass concentrations of PM_10_ were the most dispersed, and those of PM_2.5_ and PM_1_ were both concentrated. The most frequent concentration was 38.7 μg/m^3^, accounting for ~1.85%. The PM_2.5_ concentration of 24.3 μg/m^3^ was as high as 4.07%. The PM_1_ concentrations were 21.0 μg/m^3^ and 19.2 μg/m^3^, which accounted for 3.14%. In the canteen, the larger the particle size, the higher was the concentration.

**Figure 7:**
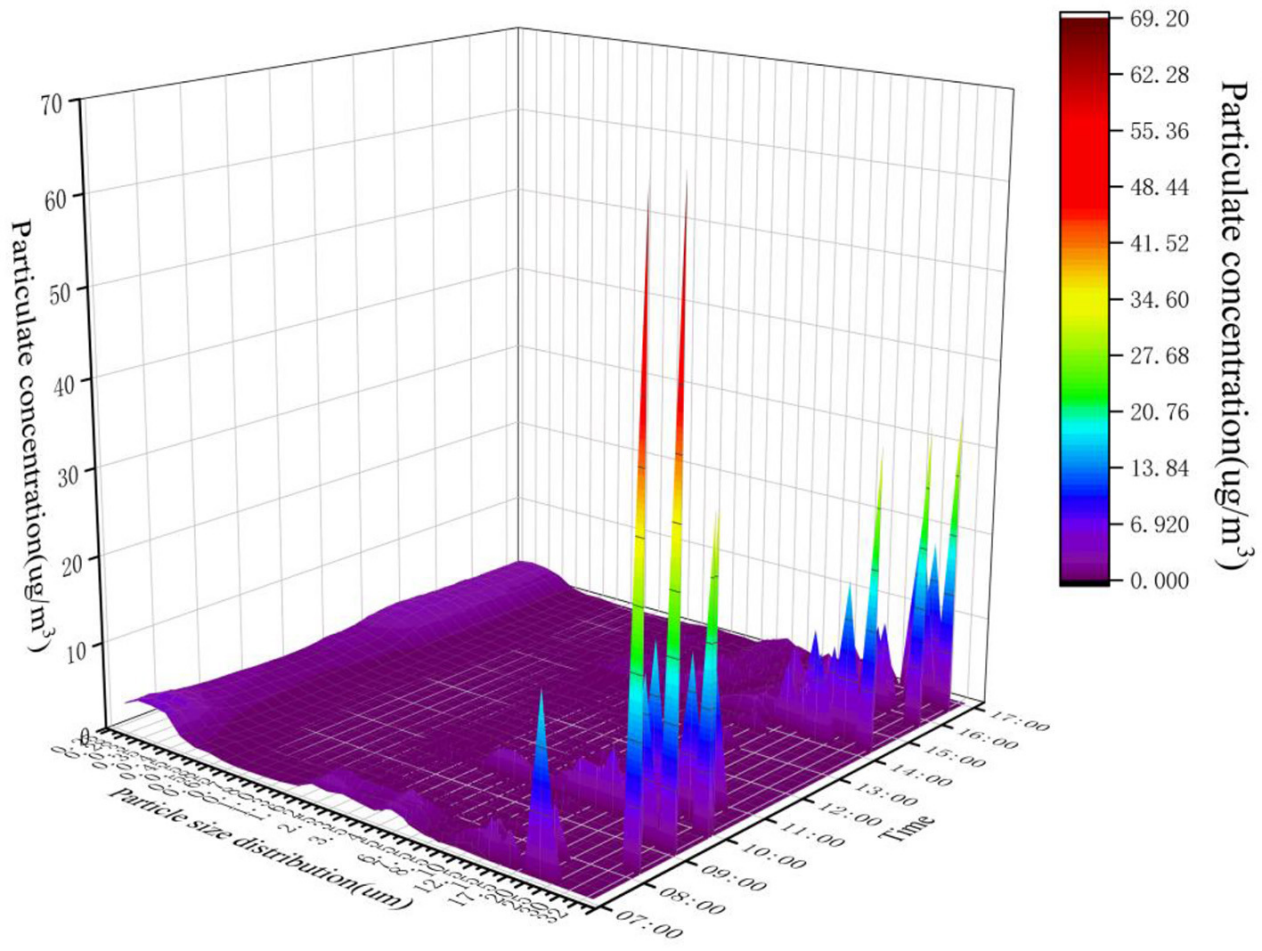
Particle diameter distributions in the library: particle diameter-time-concentration.

**Figure 8:**
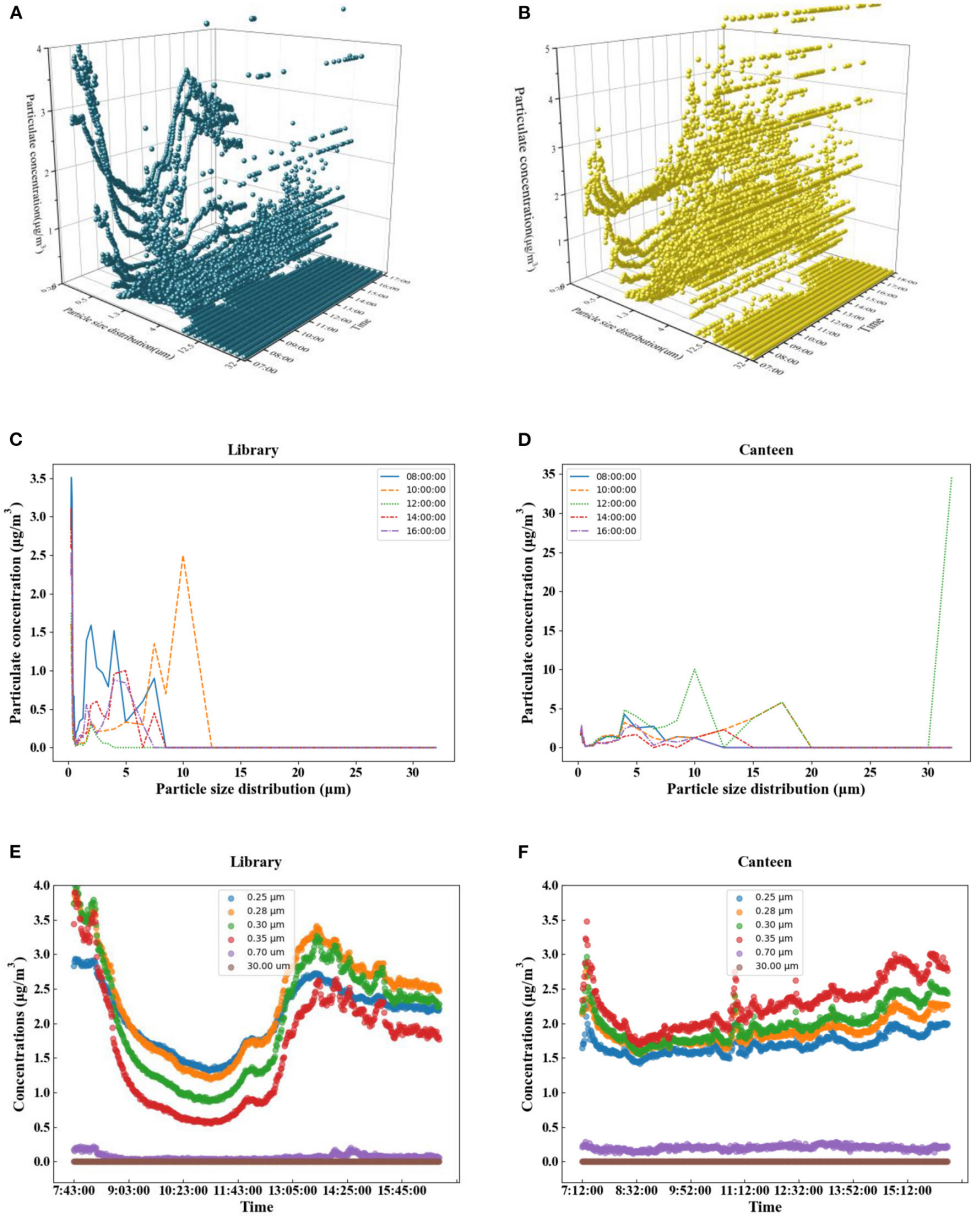
The 95% sample values particle size-time-concentration distribution for the library **(A)** and canteen **(B)**. Concentrations of various particle sizes at different times **(C–F)**.

In this study, we calculated the particle distributions of particles with different single particle sizes on the same day, and a particle diameter-time-concentration distribution map (DTC map) was drawn so that the distributions of the various parameters could be more intuitively examined. As shown in Figure 7, a small number of particles with diameters above 17.5 μm had a concentration of more than 10 μg/m^3^, while the particles with different particle sizes were mainly concentrated below 4 μg/m^3^. The particles measured at the 95% sample values in the library and canteen were analyzed as shown in Figure 8. The particle sizes below 6.5 μm in the library changed obviously, and the particle sizes above 6.5 μm were 0 or occasionally slightly fluctuated. Particles with particle sizes below 0.45 μm first decreased and then increased in the morning, and the curve changed significantly. The mass concentration of particles with particle sizes of 0.5~1.30 μm was relatively stable, ranging from 0.55 to 0.79 μg/m^3^. The concentration of particles with particle sizes of 1.6~5.0 μm changed significantly, with a range of 0.03~ 3.34 μg/m^3^. The general trend also indicated a first decrease and then an increase over time during 1 day. The concentration of particle sizes from 6.5 to 32.0 μm was occasionally prominent but almost maintained at 0 μg/m^3^ throughout the day. In the canteen, the particle concentration of 0.25~0.58 μm particle sizes was lower than 3.48 μg/ m^3^, and the concentration of 0.65–0.8 μm particle sizes was 0.17 μg/m^3^. Different from the case for the library, the particle sizes were within the range of 15~32.0 μm, so the concentrations were almost maintained at 0 μg/m^3^ all day. By comparing Figures 8C,D, it is not difficult to find that the high concentrations of particles in the library were localized within the small particle size range (0.25~0.45 μm), while the high concentrations of particles in the canteen were in the medium particle size range of 2.0~8.5 μm. The mass concentrations of large particulate matter (>15 μm) in both places were close to zero. Figures 8E,F show the time distributions of particulate matter in a day. The concentration distribution in the library was relatively regular, with a significant peak and valley period, which is consistent with the variation trend in the outdoor particulate matter concentration. The particles in the canteen were relatively dispersed during the day, and there were no periods when the particle size was too concentrated.

### Workplace Health Assessment of Airborne Particulate Matter in Public Buildings

Promulgated by the BS EN 481 standard of the British Standards Institution, air particles in the atmosphere of the workplace affect safety accordingly. Particles in the size range of 10–100 μm were considered to be inhalable particulate matter (Inhalable fraction). Those particles with sizes between 4 and 10 μm were considered to be inhalable thoracic particulate matter (Thoracic sub fraction), and particles of other sizes <4 μm belonged to alveoli particulate matter (Alveoli fraction) (57).

**Figure 9:**
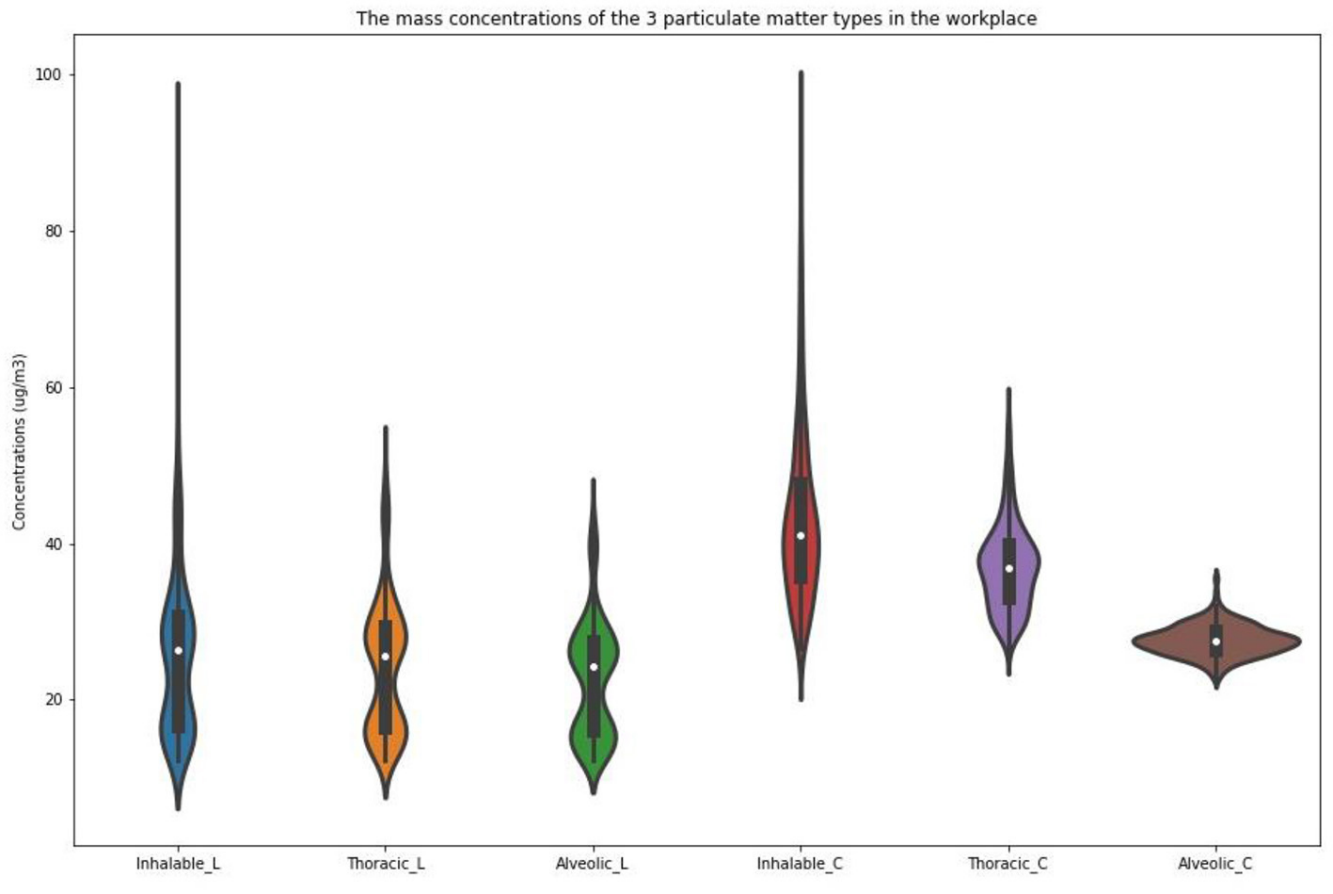
Assessment of the mass concentrations of the 3 particulate matter types in the workplace based on the BS EN 481-1993 standard.

In this test, the particle size mass concentrations of two public buildings were classified and statistically calculated. There were significant differences in the concentration of different types of particulate matter in different public buildings (*p* < 0.01, Friedman test). A box plot was added based on the violin plot to highlight the value distribution, and the result is shown in Figure 9. The gray box on the line in the middle of the fill block represents the quartile range, the fine gray line extending from it represents the 95% sample values, and the thick gray line in the center of each box represents the median. The x-coordinate is the particle classification, and the y-coordinate is the particle mass concentration. As shown in Figure 9, the canteen had the most concentrated inhalable alveoli particles, with a median mass concentration of 27.6 μg/m^3^ and an overall concentration of 26.3~28.8 μg/m^3^. The inhalable particulate matter mass concentration of the dispersed (median 41.2 μg/m^3^) and inhalable particle concentration after the inhalable thorax particulate matter pulmonary alveolus ranged between 33 and 40.03 μg/m^3^. In the library, the three kinds of workplace air-particulate-mass-concentration distributions were similar. As seen from the figure, the concentrations of particles in the library fell at both ends, and the mass concentrations of human-inhalable particles were ~16.7 and 30.7 μg/m^3^. Upon comparing the particulate matter at the two sites, the mass concentration of inhalable particulate matter in the canteen was found to be 1.52 times higher than that in the library.

**Figure 10:**
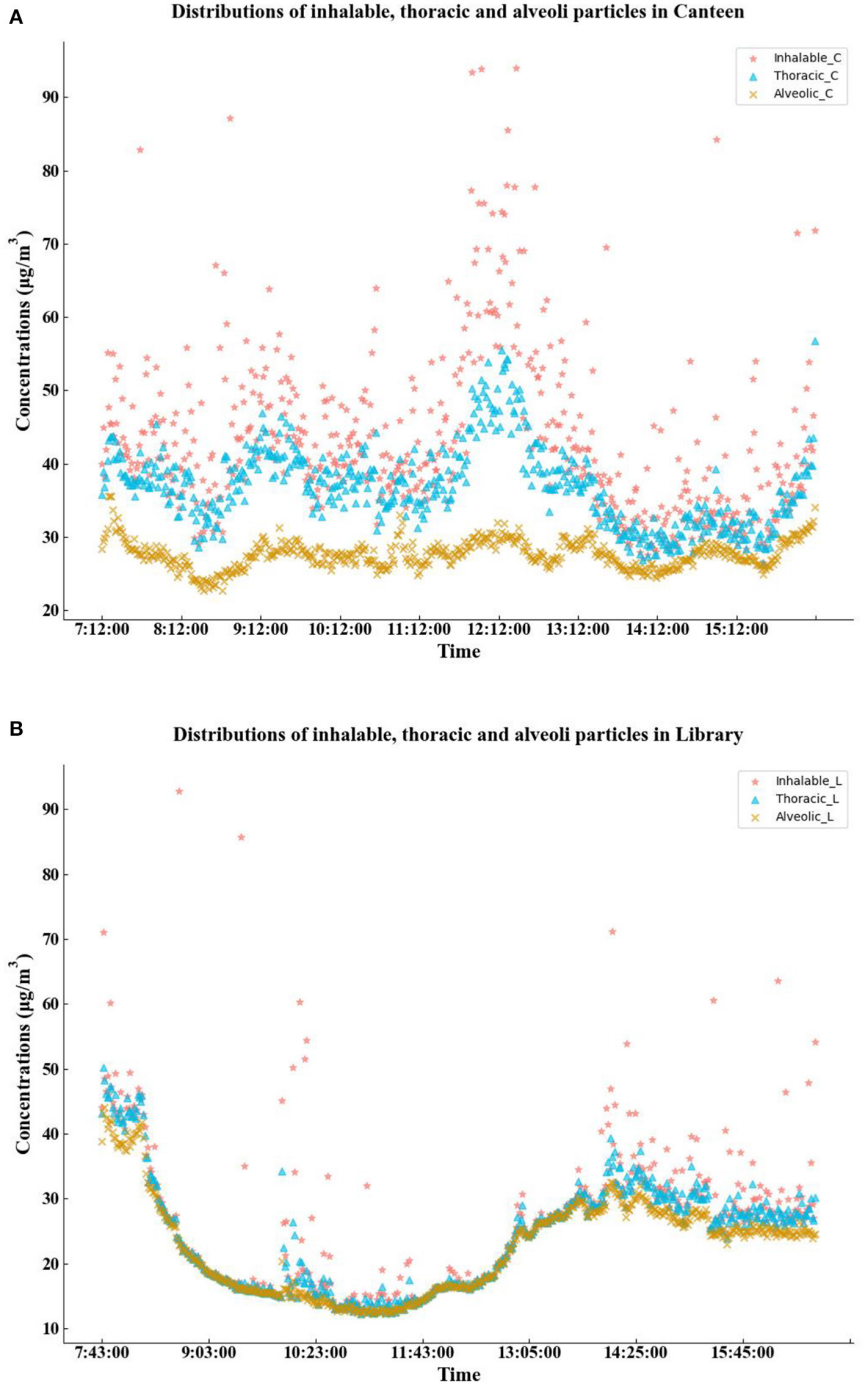
Distributions of inhalable, thoracic, and alveoli particles in the canteen and library.

The mass concentrations of particulate matter were plotted based on the time duration. As shown in Figure 10, the canteen experienced a peak in inhalable particulate matter concentrations of 209.8 μg/m^3^. At the specific time of 12:02, the pollution situation in the workplace was more serious because this point was during lunch time, and the cooking sources of particulate matter were more complicated. The mass concentration of either particle was higher than 20 μg per m^3^. Considering the BS EN 481 standard, the concentrations of particulate matter during business hours were maintained at relatively high levels. The three workplaces in the library had lower overall concentrations of particulate matter but also had extremely high levels of 92.8 and 85.7 μg/m^3^ in the morning. The concentrations of thoracic-inhalable particulate matter in the canteen ranged from 26.4 to 56.8 μg/m^3^, and the concentration of alveoli-inhalable particulate matter ranged from 22.7 to 35.6 μg/m^3^. The concentrations of all three types of particulate matter in the library began to decrease in the morning, reaching a minimum of 12.3 μg/m^3^ at 11:06 a.m., at 10:57 a.m., and 11:06 a.m. The concentrations of particulate matter at the two sites showed opposite trends at noon.

### Feasibility Analysis of the Fuzzy Comprehensive Evaluation Model

Indoor air quality, as compared to other problems, is more complex, since there is virtually no full guarantee of a comprehensive result. As a result, indoor air quality evaluation is more dependent on the uncertainty pollutants that cause the risks. Decisions made in the face of limited contaminations may lead to mistakes. Moreover, current air assessment methods do not take into account interrelationship, leading to incorrect estimates. False assessments lead to high cost or professional healthy problems.

Indoor air quality involved medicine, building environment, and architectural design, there have been many mathematical assessment models, but these models have yet to be on the air quality evaluation system to quantify the influence extent of each factor in the description, therefore, it is necessary to improve on existing models, develop a new dynamic evaluation model and give the corresponding evaluation method, for making it applicable to different places or becoming the main basis of regulating and improving air quality.

In response to these facts, it was decided to carry out this research, with the main aim of developing a fuzzy model of assessment for indoor air quality in public buildings. The model will be a useful tool to support decision-making processes, especially in professional health problems which most authorities ignore.

The results of our study will also support the further development of methodologies for assessing other pollutants, taking into account biological and radioactive contaminations, including bacterial colony and Radon (Rn).

Based on the classification standard for indoor environmental quality (58), this study combined the boundary values of each pollutant concentration and summarizes them in Table 3 (54, 59).

**Table 3:** Threshold of indoor air chemical pollutants.

**Level**	**IAQI**	**PM_**2.5**_ (mg/m^**3**^)**	**PM_**10**_(mg/m^**3**^)**	**CO (ppm)**	**O_**3**_ (ppm)**	**SO_**2**_ (ppm)**	**NO_**2**_ (ppm)**
I	50	0.010	0.033	2.0	0.002	0.04	0.07
II	100	0.019	0.055	5.5	0.01	0.07	0.11
III	150	0.038	0.091	9.3	0.05	0.13	0.29
IV	200	0.075	0.150	23.1	0.10	0.18	0.50
V	300	0.290	0.411	50	0.25	0.25	2.50

The air quality monitoring data of the four places with large amounts of people flowing through at noon were adopted, including a library (113.4019°E, 23.0439°N), a canteen (113.3904°E, 23.0485°N), a waiting hall (113.3502°E, 23.1761°N) and a department store (113.3983°E, 23.0688°N), as shown in Table 4. With the help of the MATLAB program, the maximum feature vector was calculated, and the consistency test was carried out. Finally, the sets *R*_1_ ~ *R*_4_ of the four sets of weights were obtained.

$$\begin{array}{lll} R_{1} & = & \left[\begin{array}{ccccc} \text{0} & 0.4340 & 0.5660 & 0 & 0\\ 0.9350 & 0.0650 & 0 & 0 & 0\\ 1.0000 & 0 & 0 & 0 & 0\\ 0.8750 & 0.1250 & 0 & 0 & 0\\ 1.0000 & 0 & 0 & 0 & 0\\ 1.0000 & 0 & 0 & 0 & 0 \end{array}\right]\\ R_{2} & = & \left[\begin{array}{ccccc} 0 & 0.6680 & 0.3320 & 0 & 0\\ 0.2050 & 0.7950 & 0 & 0 & 0\\ 1.0000 & 0 & 0 & 0 & 0\\ 0 & 0.7250 & 0.2750 & 0 & 0\\ 1.0000 & 0 & 0 & 0 & 0\\ 1.0000 & 0 & 0 & 0 & 0 \end{array}\right]\\ R_{3} & = & \left[\begin{array}{ccccc} 0 & 0.0247 & 0.9753 & 0 & 0\\ 0 & 0.7825 & 0.2175 & 0 & 0\\ 1.0000 & 0 & 0 & 0 & 0\\ 0 & 0.3750 & 0.6250 & 0 & 0\\ 1.0000 & 0 & 0 & 0 & 0\\ 1.0000 & 0 & 0 & 0 & 0 \end{array}\right]\\ R_{4} & = & \left[\begin{array}{ccccc} 0 & 0.8700 & 0.1300 & 0 & 0\\ 0.4427 & 0.5573 & 0 & 0 & 0\\ 0.9886 & 0.0114 & 0 & 0 & 0\\ 0 & 0.8500 & 0.1500 & 0 & 0\\ 1.0000 & 0 & 0 & 0 & 0\\ 1.0000 & 0 & 0 & 0 & 0 \end{array}\right] \end{array}$$

The membership degree of fuzzy set is used to describe the degree to which an element belongs to a fuzzy set. The larger the membership is, the higher the degree that the element belongs to the fuzzy set is, and vice versa. In some cases, the maximum membership principle will cause substantial information loss, so it cannot be objectively and effectively evaluated, and there may be one-sided results. In this paper, the *b*_j_ obtained from Table 5 was used for reference to improve the fuzzy evaluation weighted-average grade method (60, 61), and the weighted average was determined according to formula (7) to make the evaluation grade quantifiable and more intuitive.

(7) $$\begin{array}{l} J=\overset{m}{\underset{i=1}{\sum }}\left(100-20\left(j-1\right)\right)\cdot b_{\text{j}} \end{array}$$

Here, *J* is the grading value, and 0 ≤ *J* ≤ 100. Setting the separation points at four values of 50, 60, 70 and 85 corresponds to the five grade intervals. The higher was the value, the closer it was to reaching level I (i.e., 0 ≤ *J* ≤ 50, level V; 50 ≤ *J* ≤ 60, level IV; 60 ≤ *J* ≤ 70, level III; 70 ≤ *J* ≤ 85, level II; 85 ≤ *J* ≤ 100, level I). The weighted average level for calculating the level of the library value *J*_1_ was 91.536 (level I), and the level of the canteen evaluation value *J*_2_ was 86.336 (level I→level II). The level of the waiting hall evaluation value *J*_3_ was 80.378 (level II), and the level of the department store evaluation value *J*_4_ was 88.626 (level I).

**Table 4:** Air quality field test data.

**Site**	**PM_**2.5**_ (μg/m^**3**^)**	**PM_**10**_ (μg/m^**3**^)**	**CO (ppm)**	**O_**3**_ (ppb)**	**SO_**2**_ (ppb)**	**NO_**2**_ (ppb)**
Library	29.76	34.44	0.00	3.00	0.00	2.00
Canteen	25.30	50.50	0.82	21.00	0.00	8.00
Waiting hall	37.53	62.83	0.65	35.00	15.00	59.00
Department store	21.47	45.26	2.04	16.00	0.00	32.00

**Table 5:** Comprehensive evaluation vector values of different sites.

**Area**	**I**	**II**	**III**	**IV**	**V**
Library	0.7183	0.1402	0.1415	0	0
Canteen	0.4410	0.4348	0.1242	0	0
Waiting hall	0.4000	0.2189	0.3811	0	0
Department store	0.4863	0.4587	0.0550	0	0

## Conclusions

By taking contamination risks as the evaluation indexes and considering the uncertainty of the evaluation indexes and the fuzziness of threshold, the method of indoor air quality evaluation is proposed. This method can make the best of each factor to make decisions for long-term health assessments. Based on fuzzy theory, the evaluation index weight can be obtained, combining with medical literature. The results reflect the impact of valuation indicators on indoor air assessment in the evaluation system of monitoring contaminations. This model modifies the evaluation function by introducing the boundary values of each pollutant concentration, which reduces the error caused by the mutual interference in evaluation indexes to some degree. The cases of the public buildings show that the indoor air assessment model can evaluate the exposed risk of practitioners properly, and the evaluation results are in accordance with the actual condition.

By analyzing the measured data of indoor and outdoor chemical pollutant concentrations in public buildings in Guangzhou, several conclusions can be drawn as follows:

1. Indoor particulate matter and outdoor particulate matter in the library showed obvious synchronism. PM_2.5_ and PM_10_ both showed a trend of first decreasing, then increasing and finally decreasing during the test. The PM_10_ concentration in the library and canteen peaked at 07:45 in the morning and at 16:11, respectively (48.9 and 59 μg/m^3^). The PM_2.5_ concentration in the library was higher than the outdoor concentration in the period beyond 9:00~13:00, and the PM_10_ concentration in the canteen changed sharply and was frequently higher than the outdoor value. The PM_2.5_ concentrations in the canteen reached extreme values during the cooking periods before breakfast, lunch, and dinner, which were 32.5, 30.6, and 30.3 μg /m^3^, respectively. The primary indoor pollutant was particulate matter, while the primary outdoor pollutant was CO. The NO concentration in the library fluctuated more violently, and CO_2_ in the canteen peaked twice in the morning and at noon.
2. The Pearson correlation coefficient of the PM_10_ and PM_2.5_ concentrations in the library was 0.98, and PM_10_ and SO_2_ in the canteen were negatively correlated, with a correlation coefficient of −0.65. Although the Pearson correlation coefficients of the same chemical pollutants in different public buildings may vary greatly, PM_2.5_ and PM_1_ were always highly positively correlated, O_3_ in the library was weakly positively correlated with other pollutants, and PM_2.5_ and PM_1_ in the canteen were almost negatively correlated with other pollutants.
3. The largest concentrations of PM_10_ in the library were 26.5 and 16.6 μg/m^3^, accounting for 1.68% of the total data. The larger was the particle size in the canteen, the higher was the concentration observed. The concentration distribution in the library was relatively regular, with a significant peak and valley period, which was consistent with the variation trend in the outdoor particle concentration. The particles in the canteen were relatively dispersed during the day, and there were no periods when the particle size was too concentrated.
4. The high concentration of particles in the library was associated with the small particle size range (0.25~0.45 μm), while the high concentration of particles in the canteen was associated with the medium particle size range of 2.0~8.5 μm. The mass concentration of PM_10_ in the canteen was 1.52 times higher than that in the library. When choosing particle purification equipment, the particle size removal efficiency can be considered in a targeted way. The library prefers the removal of fine particles, while the canteen prefers the removal of large particles.
5. The level of the library was calculated using the fuzzy comprehensive evaluation method for level I, and the level of the waiting hall was calculated for level II. Although some sites were clean, both sites were cautious about a sharp increase in particulate matter. The pollutants in the library and the canteen in the morning mainly came from outdoors, while the PM_10_ content in the canteen at noon was relatively high; thus, the times of operating indoor air purification equipment can be concentrated during these periods. The staff should avoid staying indoors too much at peak concentration times, because at these times, the indoor pollutant concentrations were higher than the outdoor concentrations, and it is suggested that the air exchange times be strengthened and that other protective measures be taken.

Further analytical work is needed to investigate how the effect of the ultrafine particles or the submicron particles impacts other contamination in buildings and any potential changes in health impacts. Additionally, systematic measurements are needed to separate the impacts of VOCs from different public spaces on general population health and source control, to raise awareness of the potential impacts of indoor pollutants. These chemicals are subject to reactions with other species, as such research to suggest optimal strategies considering all indoor pollutants present are needed. Therefore, the evidence comes down to the need for health-based guideline values for as much contamination as possible, rather than an individual pollutant limit value.

## Data Availability Statement

The raw data supporting the conclusions of this article will be made available by the authors, without undue reservation.

## Ethics Statement

Written informed consent was obtained from the individual(s) for the publication of any potentially identifiable images or data included in this article.

## Author Contributions

XL conceived of the study, designed and completed the experiments, participated in data analysis and interpretation, prepared figures, and wrote the manuscript. ZL assisted in writing review, editing, and performing the analysis with constructive discussions. HZ helped perform the experiments and contributed conceptually to the project. XH analyzed the data and provided the software for completing the work. All authors read and approved the final manuscript.

## Conflict of Interest

The authors declare that the research was conducted in the absence of any commercial or financial relationships that could be construed as a potential conflict of interest.

## References

[1] Morawska L Afshari A Bae GN Buonanno G Chao CYH Hanninen O. Indoor aerosols: from personal exposure to risk assessment. Indoor Air. (2013) 23:462–87. 10.1111/ina.12044 23574389

[2] Klepeis NE Nelson WC Ott WR Robinson JP Tsang AM Switzer P. The National Human Activity Pattern Survey (NHAPS): a resource for assessing exposure to environmental pollutants. J Exp Sci Environ Epidemiol. (2001) 11:231–52. 10.1038/sj.jea.7500165 11477521

[3] Brasche S Bischof W. Daily time spent indoors in German homes – Baseline data for the assessment of indoor exposure of German occupants. (2005). 10.1016/j.ijheh.2005.03.003 16078638

[4] Bernstein JA Alexis N Bacchus H Bernstein IL Fritz P Horner E. The health effects of nonindustrial indoor air pollution. J Allergy Clin Immunol. (2008) 121:585–91. 10.1016/j.jaci.2007.10.045 18155285

[5] Dobbin NA Sun L Wallace L Kulka R You H Shin T. The benefit of kitchen exhaust fan use after cooking - An experimental assessment. Build Environ. (2018) 135:286–96. 10.1016/j.buildenv.2018.02.039

[6] Logue JM Klepeis NE Lobscheid AB Singer BC. Pollutant exposures from natural gas cooking burners: a simulation-based assessment for Southern California. Environ Health Perspect. (2014) 122:43–50. 10.1289/ehp.1306673 24192135 PMC3888569

[7] Yadav IC Devi NL Li J Zhang G. Occurrence and source apportionment of halogenated flame retardants in the indoor air of Nepalese cities: implication on human health. Atmospheric Environ. (2017) 161:122–31. 10.1016/j.atmosenv.2017.04.031

[8] Dong G-H. Perspective for future research direction about health impact of ambient air pollution in China. Adv Exp Med Biol. (2017) 1017:263–8. 10.1007/978-981-10-5657-4_12 29177967

[9] Amoatey P Omidvarborna H Baawain MS Al-Mamun A. Indoor air pollution and exposure assessment of the gulf cooperation council countries: a critical review. Environ Int. (2018) 121:491–506. 10.1016/j.envint.2018.09.043 30286426 PMC7132391

[10] Hu T Jia W Cao J Huang R Li H Liu S. Indoor air quality at five site museums of Yangtze River civilization. Atmospheric Environ. (2015) 123:449–54. 10.1016/j.atmosenv.2015.10.022

[11] Sciences IAOIA. Deadly household pollution: a call to action. Indoor Air. (2006) 16:2–3. 10.1111/j.1600-0668.2005.00416.x 16420491

[12] Danesh Yazdi M Wang Y Di Q Zanobetti A Schwartz J. Long-term exposure to PM2.5 and ozone and hospital admissions of Medicare participants in the Southeast USA. Environ Int. (2019) 130:104879. 10.1016/j.envint.2019.05.073 31238267 PMC7751740

[13] Huang J Song Y Chu M Dong W Miller MR Loh M. Cardiorespiratory responses to low-level ozone exposure: the in Door Ozone Study in childrEn (DOSE). Environ Int. (2019) 131:105021. 10.1016/j.envint.2019.105021 31349208

[14] Franck U Herbarth O Röder S Schlink U Borte M Diez U. Respiratory effects of indoor particles in young children are size dependent. Sci Total Environ. (2011) 409:1621–31. 10.1016/j.scitotenv.2011.01.001 21316080

[15] Norbäck D Lu C Zhang Y Li B Zhao Z Huang C. Onset and remission of childhood wheeze and rhinitis across China — Associations with early life indoor and outdoor air pollution. Environ Int. (2019) 123:61–9. 10.1016/j.envint.2018.11.033 30496983

[16] Oliveira M Slezakova K Delerue-Matos C Pereira MC Morais S. Children environmental exposure to particulate matter and polycyclic aromatic hydrocarbons and biomonitoring in school environments: a review on indoor and outdoor exposure levels, major sources and health impacts. Environ Int. (2019) 124:180–204. 10.1016/j.envint.2018.12.052 30654326

[17] Tavera Busso I Mateos AC Juncos LI Canals N Carreras HA. Kidney damage induced by sub-chronic fine particulate matter exposure. Environ Int. (2018) 121:635–42. 10.1016/j.envint.2018.10.007 30316178

[18] Stowell JD Geng G Saikawa E Chang HH Fu J Yang C-E. Associations of wildfire smoke PM2.5 exposure with cardiorespiratory events in Colorado 2011–2014. Environ Int. (2019) 133:105151. 10.1016/j.envint.2019.105151 31520956 PMC8163094

[19] da Costa e Oliveira JR Base LH de Abreu LC Filho CF Ferreira C Morawska L. Ultrafine particles and children's health: literature review. Paediatr Respir Rev. (2019) 32:73–81. 10.1016/j.prrv.2019.06.003 31427160

[20] Tang X Bai Y Duong A Smith MT Li L Zhang L. Formaldehyde in China: production, consumption, exposure levels, and health effects. Environ Int. (2009) 35:1210–24. 10.1016/j.envint.2009.06.002 19589601

[21] Sarigiannis DA Karakitsios SP Gotti A Liakos IL Katsoyiannis A. Exposure to major volatile organic compounds and carbonyls in European indoor environments and associated health risk. Environ Int. (2011) 37:743–65. 10.1016/j.envint.2011.01.005 21354626

[22] Buoli M Grassi S Caldiroli A Carnevali GS Mucci F Iodice S. Is there a link between air pollution and mental disorders? Environ Int. (2018) 118:154–68. 10.1016/j.envint.2018.05.044 29883762

[23] Kioumourtzoglou MA Power MC Hart JE Okereke OI Coull BA Laden F. The association between air pollution and onset of depression among middle-aged and older women. Am J Epidemiol. (2017) 185:801–9. 10.1093/aje/kww163 28369173 PMC5411676

[24] Andersen ZJ Stafoggia M Weinmayr G Pedersen M Galassi C Jørgensen JT. Long-term exposure to ambient air pollution and incidence of postmenopausal breast cancer in 15 European Cohorts within the ESCAPE Project. Environ Health Perspect. (2017) 125:919. 10.1289/EHP1742 29033383 PMC5933325

[25] Andersen ZJ Ravnskjaer L Andersen KK Loft S Brandt J Becker T. Long-term exposure to fine particulate matter and breast cancer incidence in the Danish Nurse Cohort Study. Cancer Epidemiol Biomarkers Prevent. (2016) 26:428–30. 10.1158/1055-9965.EPI-16-0578 27913396

[26] Goldberg MS Villeneuve PJ Crouse D To T Weichenthal SA Wall C. Associations between incident breast cancer and ambient concentrations of nitrogen dioxide from a national land use regression model in the Canadian National Breast Screening Study. Environ Int. (2019) 133:105182. 10.1016/j.envint.2019.105182 31648153

[27] Atkinson RW Carey IM Kent AJ van Staa TP Anderson HR Cook DG. Long-term exposure to outdoor air pollution and incidence of cardiovascular diseases. Epidemiology. (2013) 24:44–53. 10.1097/EDE.0b013e318276ccb8 23222514

[28] Li X Yi H Wang H. Sulphur dioxide and arsenic affect male reproduction via interfering with spermatogenesis in mice. Ecotoxicol Environ Safety. (2018) 165:164–73. 10.1016/j.ecoenv.2018.08.109 30195209

[29] Zhang J Li Z Qie M Zheng R Shetty J Wang J. Sodium fluoride and sulfur dioxide affected male reproduction by disturbing blood-testis barrier in mice. Food Chem Toxicol. (2016) 94:103–11. 10.1016/j.fct.2016.05.017 27237588

[30] Shrubsole C Dimitroulopoulou S Foxall K Gadeberg B Doutsi A. IAQ guidelines for selected volatile organic compounds (VOCs) in the UK. Build Environ. (2019) 165:106382. 10.1016/j.buildenv.2019.106382

[31] Azuma K Jinno H Tanaka-Kagawa T Sakai S. Risk assessment concepts and approaches for indoor air chemicals in Japan. Int J Hygiene Environ Health. (2020) 225:113470. 10.1016/j.ijheh.2020.113470 32050149

[32] Fanger PO. Introduction of the olf and the decipol units to quantify air pollution perceived by humans indoors and outdoors. Energy Build. (1988) 12:1–6. 10.1016/0378-7788(88)90051-5

[33] Thach T-Q Tsang H Cao P Ho L-M. A novel method to construct an air quality index based on air pollution profiles. Int J Hygiene Environ Health. (2018) 221:17–26. 10.1016/j.ijheh.2017.09.012 28988894

[34] Zhu C Li N. Study on Grey Clustering Model of Indoor Air Quality Indicators. Proc Eng. (2017) 205:2815–22. 10.1016/j.proeng.2017.09.895

[35] Quang TN He C Morawska L Knibbs LD. Influence of ventilation and filtration on indoor particle concentrations in urban office buildings. Atmospheric Environ. (2013) 79:41–52. 10.1016/j.atmosenv.2013.06.009

[36] Kim S-S Kang D-H Choi D-H Yeo M-S Kim K-W. Comparison of strategies to improve indoor air quality at the pre-occupancy stage in new apartment buildings. Build Environ. (2008) 43:320–8. 10.1016/j.buildenv.2006.03.026

[37] Lin BY Huangfu YB Lima N Jobson B Kirk M O'Keeffe P. Analyzing the relationship between human behavior and indoor air quality. J Sens Actuar Netw. (2017) 6:18. 10.3390/jsan6030013

[38] Luo N Weng W Xu X Hong T Fu M Sun K. Assessment of occupant-behavior-based indoor air quality and its impacts on human exposure risk: a case study based on the wildfires in Northern California. Sci Total Environ. (2019) 686:1251–61. 10.1016/j.scitotenv.2019.05.467 31412521

[39] Brantley HL Hagler GSW Kimbrough ES Williams RW Mukerjee S Neas LM. Mobile air monitoring data-processing strategies and effects on spatial air pollution trends. Atmospheric Measur Tech. (2014) 7:2169–83. 10.5194/amt-7-2169-2014

[40] Bossche JVD Peters J Verwaeren J Botteldooren D Theunis J Baets BDJAE. Mobile monitoring for mapping spatial variation in urban air quality: development and validation of a methodology based on an extensive dataset. Atmospheric Environ. (2015) 105:148–61. 10.1016/j.atmosenv.2015.01.017

[41] Aihua H Zhongliang D Yao Z editors. Study on the method of indoor wireless channel characteristic measurement and analysis based on Vector Network Analyzer. In: 13th IEEE International Conference on Electronic Measurement & Instruments (ICEMI). (2017). 10.1109/ICEMI.2017.8265965

[42] Pei Y Guo M. The fundamental principle and application of sliding average method. Gun Launch Control. (2001) 1:21–3.

[43] Zhao C Chen J Du P Yuan H. Characteristics of climate change and extreme weather from 1951 to 2011 in China. Int J Environ Res Public Health. (2018) 15:2540. 10.3390/ijerph15112540 30428540 PMC6265753

[44] Liu GR Nguyen TT. Smoothed Finite Element Methods. Boca Raton, FL: Taylor & Francis (2010).

[45] Ziemba PJE. Multi-criteria fuzzy evaluation of the planned offshore wind farm investments in Poland. Energies. (2021) 14:978. 10.3390/en14040978

[46] Chen JF Hsieh HN Do QH. Evaluating teaching performance based on fuzzy AHP and comprehensive evaluation approach. Appl Soft Comput. (2015) 28:100–8. 10.1016/j.asoc.2014.11.050

[47] Lin Y Lin C Qiu X. Fuzzy Comprehensive evaluation method of masonry structure safety based on grey clustering theory. Math Problems Eng. (2018) 2018:10192. 10.1155/2018/8710192

[48] Zhao H Yao L Mei G Liu T Ning Y. A fuzzy comprehensive evaluation method based on AHP and entropy for a landslide susceptibility map. Entropy. (2017) 19:396. 10.3390/e19080396

[49] Javid A Hamedian AA Gharibi H Sowlat MH. Towards the application of Fuzzy Logic for Developing a Novel Indoor Air Quality Index (FIAQI). Iranian J Public Health. (2016) 45:203–13. 27114985 PMC4841875

[50] Sowlat MH Gharibi H Yunesian M Mahmoudi MT Lotfi S. A novel, fuzzy-based air quality index (FAQI) for air quality assessment. Atmospheric Environ. (2011) 45:2050–9. 10.1016/j.atmosenv.2011.01.060

[51] Long L Li ZD. An assessment model of monitoring risk in deep excavation based on fuzzy theory. Indoor Built Environ. (2020) 29:221–9. 10.1177/1420326X19856671

[52] Onkal-Engin G Demir I Hiz H. Assessment of urban air quality in Istanbul using fuzzy synthetic evaluation. Atmospheric Environ. (2004) 38:3809–15. 10.1016/j.atmosenv.2004.03.058

[53] Feng N Fu B. Indoor air quality evaluation method and its mathematical model. J Suzhou Univ Sci Technol. (2015) 32:9–14.

[54] Ming L lin L chang S wenbo D. The study on the theory of grade division of fuzzy comprehensive evaluation of indoor environment. Low Temp Architect Technol. (2018) 40:109–24.

[55] Xiao K Yuku W Guang W Bin F Yuanyuan Z. Spatiotemporal Characteristics of Air Pollutants (PM10, PM2.5, SO2, NO2, O3, and CO) in the Inland Basin City of Chengdu, Southwest China. Atmosphere. (2018) 9:74. 10.3390/atmos9020074

[56] Du B Gao J Chen J Stevanovic S Ristovski Z Wang L. Particle exposure level and potential health risks of domestic Chinese cooking. Build Environ. (2017) 123:564–74. 10.1016/j.buildenv.2017.07.031

[57] Standards BSIB. Workplace Atmospheres. Size Fraction Definitions for Measurement of Airborne Particles. London: BSI British Standards (1993).

[58] Ting-yun H. Development of Indoor-Vehicle Air Quality Index System. Taipei: National Taipei University of Technology (2003).

[59] Zhu C. Reasearch on Comfort Evaluation and Grey Theory Analysis of Indoor Environment. Changsha: Hunan University (2012).

[60] Zadeh MA Sadrania A Zibandeh M Rostami P editors. Determining a suitable location for a Sewage Treatment Plant using a new fuzzy weighted average (FWA) method based on left and right scores. In: 13th Iranian Conference. Qazvin: IEEE (2013).

[61] Jie Z Xin-ze L Ai-ying K Yuan H Guo-quan Z. Application of fuzzy synthetic evaluation method in nanxi river water quality evaluation. Zhejiang Hydrotech. (2018) 46:8–28.

