# Supplementary material for: Study of the Characteristics and Comprehensive Fuzzy Assessment of Indoor Air Chemical Contamination in Public Buildings
Source: Front Public Health. 2021 May 5;9:579299. doi: 10.3389/fpubh.2021.579299 (PMC8138320; doi:10.3389/fpubh.2021.579299)
Supplement: Supplementary file 2 [file Data_Sheet_2.doc]

**Appendix:**

***Variations of outdoor pollutant concentration***

On the basis of literature (1, 2), there is a strong correlation between indoor and outdoor pollutants. And hourly variations of pollutant concentrations are shown in Figure A1. The characteristics of pollutants could be better understood by analyzing the concentration difference between indoor and outdoor.

|  |  |
| --- | --- |

**Figure A1.** Hourly variations of pollutant concentrations at Haizhuhu (Left, 2019.04.16) and Chisha (Right, 2019.04.15) air monitoring stations.

***Variations of indoor pollutant concentration***

Taking 09:00 am as an example, the I/O ratio of CO was 1.25, the I/O ratio of NO_2_ was 0.11, the I/O ratio of O_3_ was 1.43, and the I/O ratio of SO_2_ was 1.07 (Figure A2). These amounts were still higher than the outdoor concentrations, although the indoor chemical pollutants did not exceed the standard limits.

|  |  |
| --- | --- |

**Figure A2.** Hourly variations of chemical pollutants in the library (Left) and canteen (Right).

Figure A3 and Figure A4 show the hourly variations in the percentages of 6 gases in the two locations.

|  |
| --- |

**Figure A3.** Hourly variations of the other 6 gases in the library.

|  |
| --- |

**Figure A4.** Hourly variations of the other 6 gases in the canteen.

***Example calculation***

Taking library as an example, calculation process could be as follows:

In library, c(PM_2.5_) =29.76 μg/m^3^; c(PM_10_) =34.44 μg/m^3^; c(CO) =0.00 ppm; c(O_3_) =3.00 ppb; c(SO_2_) =0.00ppb; c(NO_2_) =2.00 ppb.

A membership function can be calculated by using Formula (3)-(5):

According to Table 3, for PM_2.5_ (mg/m^3^), *u*_i_= *u*_PM2.5_ =0.02976, *s*_1_=0.01, *s*_2_=0.019, *s*_3_=0.038, *s*_4_=0.075, *s*_5_=0.290.

$$r_{11}\text{=}0,u_{\text{PM}_{2.5}}\text{=0.2976}>s_{2}\text{=0.019}$$

$$r_{12}\text{=}\frac{s_{3}-u_{\text{PM}_{2.5}}}{s_{3}-s_{2}}\text{=}\frac{\text{0.038-0.02976}}{0.038-0.019}\approx0.4340,0.019<0.02976<0.038$$

$$r_{13}\text{=}\frac{u_{\text{PM}_{2.5}}-s_{2}}{s_{3}-s_{2}}\text{=}\frac{0.02976-0.019}{0.038-0.019}\approx0.5660,0.019<0.02976<0.038$$

$$r_{14}\text{=}0,u_{\text{PM}_{2.5}}=0.02976<s_{3}=0.038$$

$$r_{15}\text{=}0,u_{\text{PM}_{2.5}}=0.02976<s_{4}=0.075$$

For PM_10_ (mg/m^3^), *u*_i_= *u*_PM10_ =0.03444, *s*_1_=0.033, *s*_2_=0.055, *s*_3_=0.091, *s*_4_=0.150, *s*_5_=0.411.

$$r_{21}\text{=}\frac{s_{2}-u_{\text{PM}_{10}}}{s_{2}-s_{1}}\text{=}\frac{\text{0.055-0.03444}}{0.055-0.033}\approx0.9350,0.033<0.03444<0.055$$

$$r_{22}\text{=}\frac{u_{\text{i}}-s_{1}}{s_{2}-s_{1}}\text{=}\frac{\text{0.03444-0.033}}{0.055-0.033}\approx0.0650,0.033<0.03444<0.055$$

$$r_{23}\text{= 0},0.03444<0.055$$

$$r_{24}\text{= 0},0.03444<0.091$$

$$r_{25}\text{= 0},0.03444<0.150$$

For CO (ppm), *u*_i_= *u*_CO_ =0.00, *s*_1_=2.0, *s*_2_=5.5, *s*_3_=9.3, *s*_4_=23.1, *s*_5_=50.

$$r_{31}\text{= 1},0.00<2.0$$

$$r_{32}\text{= 0},0.00<2.0$$

$$r_{33}\text{= 0},0.00<5.5$$

$$r_{34}\text{= 0},0.00<9.3$$

$$r_{35}\text{= 0},0.00<23.1$$

For O_3_ (ppm), *u*_i_= *u*_O3_ =0.0030, *s*_1_=0.002, *s*_2_=0.01, *s*_3_=0.05, *s*_4_=0.10, *s*_5_=0.25.

$$r_{41}\text{=}\frac{s_{2}-u_{\text{O}_{3}}}{s_{2}-s_{1}}\text{=}\frac{\text{0.01-0.003}}{0.01-0.002}\approx0.875,0.002<0.003<0.01$$

$$r_{42}\text{=}\frac{u_{\text{O}_{3}}-s_{1}}{s_{2}-s_{1}}\text{=}\frac{\text{0.003-0.002}}{0.01-0.002}\approx0.125,0.002<0.003<0.01$$

$$r_{43}\text{= 0},0.003<0.01$$

$$r_{44}\text{= 0},0.003<0.05$$

$$r_{45}\text{= 0},0.003<0.10$$

For SO_2_ (ppm), *u*_i_= *u*_SO2_ =0.00, *s*_1_=0.04, *s*_2_=0.07, *s*_3_=0.13, *s*_4_=0.18, *s*_5_=0.25.

$$r_{51}\text{= 1},0.00<0.04$$

$$r_{52}\text{= 0},0.00<0.04$$

$$r_{53}\text{= 0},0.00<0.07$$

$$r_{54}\text{= 0},0.00<0.13$$

$$r_{55}\text{= 0},0.00<0.18$$

For NO_2_ (ppm), *u*_i_= *u*_NO2_ =0.002, *s*_1_=0.07, *s*_2_=0.11, *s*_3_=0.29, *s*_4_=0.50, *s*_5_=2.50.

$$r_{61}\text{= 1},0.002<0.07$$

$$r_{62}\text{= 0},0.00<0.11$$

$$r_{63}\text{= 0},0.00<0.11$$

$$r_{64}\text{= 0},0.00<0.29$$

$$r_{65}\text{= 0},0.00<0.50$$

∵*R*_i_=(*r*_i1_, *r*_i2_..., *r*_in_)^T^

∴$R_{1}=\left[\begin{matrix} 0 & 0.4340 & 0.5660 & 0 & 0 \\ 0.9350 & 0.0650 & 0 & 0 & 0 \\ 1.0000 & 0 & 0 & 0 & 0 \\ 0.8750 & 0.1250 & 0 & 0 & 0 \\ 1.0000 & 0 & 0 & 0 & 0 \\ 1.0000 & 0 & 0 & 0 & 0 \end{matrix}\right]$

*A*= [*a*_1_, *a*_2_,..., *a*_5_] = [0.25, 0.2, 0.2, 0.15, 0.1, 0.1]

∵$B=\left[a_{1}\text{ }a_{2}\text{ }\cdots\text{ }a_{\text{m}}\right]\left[\begin{matrix} r_{11} & r_{12} & \cdots& r_{\text{ln}} \\ r_{21} & r_{22} & \cdots& r_{2\text{n}} \\ \vdots& \vdots& \ddots& \vdots\\ r_{\text{m1}} & r_{\text{m}2} & \cdots& r_{\text{mn}} \end{matrix}\right]=\left(b_{1}\text{ }b_{2}\text{ }\cdots\text{ }b_{\text{n}}\right)=\left[\begin{matrix} 0.7183 & 0.1402 & \begin{matrix} \begin{matrix} 0.1415 & 0 \end{matrix} & 0 \end{matrix} \end{matrix}\right]$

Therefore, comprehensive evaluation vector values could be calculated:

| **Area** | **Ⅰ** | **Ⅱ** | **Ⅲ** | **Ⅳ** | **Ⅴ** |
| --- | --- | --- | --- | --- | --- |
| Library | 0.7183 | 0.1402 | 0.1415 | 0 | 0 |

Similarly, other places’ comprehensive evaluation vector values could be calculated in the same way.
